# Emergency Medicine Clerkship Grading Scheme, Grade, and Rank-List Distribution as Reported on Standardized Letters of Evaluation

**DOI:** 10.5811/westjem.18687

**Published:** 2024-10-29

**Authors:** Alexandra Mannix, Thomas Beardsley, Thomas Alcorn, Morgan Sweere, Michael Gottlieb

**Affiliations:** * University of Florida College of Medicine – Jacksonville, Department of Emergency Medicine, Jacksonville, Florida; † Rush University Medical Center, Department of Emergency Medicine, Chicago, Illinois

## Abstract

**Background:**

The Standardized Letter of Evaluation (SLOE) is a crucial component of the emergency medicine (EM) application process. Given the critical role of the SLOE, we attempted to better understand the grading scales used, as well as the distribution of grades and rank-list positions.

**Objectives:**

Our primary objective in this study was to determine the distribution of grading formats, grades given, and rank-list positions across EM clerkships using the SLOE.

**Methods:**

We performed a cross-sectional study of the grading formats, grades given, and ranking distributions as reported on the SLOE during the 2022–23 application cycle. We obtained data on SLOEs from EM residency programs accredited by the Accreditation Council for Graduate Medical Education by reviewing all applicants who applied to either of two EM residency programs in geographically different regions. Trained abstractors recorded the following data: number of students rotating in the prior year; grading format used; and grade and rank distribution among students.

**Results:**

We included 264 programs in our final analysis, after 13 programs met exclusion criteria. The majority of programs (72.2%) use an Honors/High Pass/Pass/Fail grading scheme. We determined the mean percent of each grade: Honors/A 27.6%; High Pass/B 31.1%; Pass/C 40.8%; Low Pass/D 0.2%; and Fail/F 0.3%. Finally, we determined the mean percent for each rank-list position: top 10% was 17.6%; top third 36.5%; mid third 34.1%; and low third 11.8%.

**Conclusion:**

We determined the grading schemes and grade and rank-list distributions for EM programs during the 2022–2023 academic year. Most programs used a Honors/High Pass/Pass/Fail grading scheme, with the majority of students receiving Honors or High Pass, while 0.3% failed their rotation. Both grades and rank list demonstrated evidence of a skewed distribution toward higher grades and rank-list position.

## INTRODUCTION

The Standardized Letter of Evaluation (SLOE) is a crucial part of the National Resident Matching Program (Match) process into emergency medicine (EM) residencies in the United States since it replaced the narrative letter of recommendation in the 1990s.^1^ The SLOE uses evaluations from EM rotations to compare medical students in a standardized, objective manner for the Match. The SLOEs have become universally adopted after being deemed a reliable tool in stratifying EM residency candidates, making it a key portion of the application process, especially considering recent changes to qualifying board exams moving to pass/fail.^2^

The top three facets of the application used by program directors overall are core clerkship grades, EM clerkship grades, and letters of recommendation.^2^ The EM clerkship grading is irregular, with no standardization between programs regarding the assignment of grades.^3^ Schools have a number of different grading systems as well as requirements to meet each grade level, making it more difficult to compare students from different medical schools. The clerkship grading is considered to be not as fruitful about the applicant’s overall performance as the global assessment section of the SLOE.^4^ In fact, the initial creation of the SLOE was spurred partially to combat speculated grade inflation among programs. Even so, the Council of Residency Directors in Emergency Medicine (CORD) Task Force found that grade inflation was still a limiting factor of the SLOE, with some improvement in rank-list inflation noted between 2012 and 2017.^2^ ^,^ ^4^

Many SLOE writers have indicated that they did not receive formal training in how to properly fill out the SLOE and often do not access the CORD guidelines for doing so.^5^ The creation of the eSLOE in 2016 contributed to an overall improvement in the ranking distribution of students; however, the system is still inconsistent.^4^ There is also variability in the interpretation of the SLOE, with a significant number of program directors agreeing that their interpretation of the data is impacted by who the letter writer is.^6^ Speculation exists regarding the validity of these evaluation letters because of this inconsistency, even extending to other specialties.^7^

Considering the critical role of the SLOE as a tool for rank-list determination, there is a need to better understand distribution of grades and rank-list positions. Our primary objective in this study was to determine the distribution of grade scheme, grade, and rank-list positions across EM programs using the SLOE.

## METHODS

### Study Design

We performed a cross-sectional study of the grading and ranking distributions of EM medical student clerkships as reported on the SLOE during the 2022–23 application cycle. The SLOEs were evaluated for the reported clerkship grading and rank-list positions from the previous year, a required component of the SLOE. We did not review the grades and rank-list positions that were given to current applicants who applied to our programs. This study was deemed exempt by the institutional review boards at the University of Florida – Jacksonville, and Rush University Medical Center.

### Study Protocol

We included all EM residency programs across the United States that were accredited by the Accreditation Council for Graduate Medical Education. To obtain SLOEs from each EM US residency-based clerkship, we reviewed SLOEs from all applicants during the 2022–23 cycle who applied to either of two EM residency programs that are located in different geographic regions. The University of Florida – Jacksonville is a three-year, county EM program located in Jacksonville, FL. Rush University Medical Center is a three-year, academic EM program in Chicago, IL. We included all clerkship SLOEs from programs that had an affiliated EM residency program and had reported either grade distribution or rank distribution from the prior year. We excluded programs without a rotation the preceding year, programs without reported data for the preceding year, or programs for which we did not have access to a SLOE.

### Measures

Trained abstractors from each institution collected data using a pre-piloted standardized data-abstraction tool. The abstractors recorded the following data: number of students rotating in the prior year; grading format used (eg, Honors/High Pass/Pass/Fail [H/HP/P/F]; A/B/C/D/F; P/F; other); prior year grade distribution; and prior year rank distribution (top 10%/top third/middle third/bottom third). We performed dual extraction for all programs with at least two SLOEs available.

### Data Analysis

Descriptive statistics are reported for the type of grading format and distribution. We report the mean with standard deviation and median with interquartile range (IQR) for each grade and rank distribution at the rotation level. We also report the overall reported number and reported percentage of total applicants receiving each category across all combined data. Reported number was defined as the total number of students receiving a specific grade or rank by a given program. We defined reported percentage as the percentage of the total students in a given program receiving a specific grade or rank. All analyses were performed using Microsoft Excel 2018 (Microsoft Corporation, Redmond, WA).

## RESULTS

Of 277 programs identified, we included 264 (95.3%) EM residency-based clerkships in our analysis. Thirteen clerkships met exclusion criteria (two that had no rotation the prior year, one that did not report data due to new SLOE format, and 10 with no SLOE available in our set). We identified a median of 21 (IQR 15–30) SLOEs written per program.

The majority of programs, 72.2% (190/263), used an H/HP/P/F grading scheme, followed by P/F 17.5% (46/263), A/B/C/D/F 2.7% (7/263), and other 7.6% (20/263). The other grading schemes are included in Appendix 1. One program did not provide a grading scheme. When evaluating grade distribution, we determined the mean percentage of each grade for all 14,562 students: Honors/A 27.6% (4,296); High Pass/B 31.1% (4,837); Pass/C 40.8% (5,343); Low Pass/D 0.2% (37); and Fail/F 0.3% (49). Grade distributions were then divided into non-P/F programs, and P/F programs, and presented in Table 1.

**Table 1.** Number and percentage distribution of students for each grade for various grading formats at non-pass/fail and pass/fail programs.

	Non-pass/fail programs (N = 13,599) n (%)	Pass/fail programs (N = 1,964) n (%)	All programs (N = 15,563) n (%)
Honors/A	4,296 (31.6%)		4,296 (27.6%)
High pass/B	4,837 (35.6%)		4,837 (31.1%)
Pass/C	4,380 (32.2%)	1,963 (99.9%)	6,343 (40.8%)
Low pass/D	37 (0.3%)		37 (0.2%)
Fail/F	48 (0.4%)	1 (<0.1%)	48 (0.3%)

When evaluating rank-list distribution, we determined the mean percentage for each rank-list position for all students across all programs. The mean percentages of students (6,221) for each rank-list position were as follows: in the top 10% there were 1,094 students (17.6%); in the top third there were 2,271 students (36.5%); in the mid third 2,123 students (34.1%); and in the low third 733 students (11.8%).

Finally, to assess program-level differences, we determined the number and percentage of students receiving a given grade and rank by each program. We then calculated the mean and median number and mean and median percentage across programs. The mean percentage of students given Honors across programs was 26.9%, followed by 30.7% who were given High Pass, and 41.7% Pass (Table 2). The mean percentage of students ranked in the top 10% by programs was 19.8%, followed by 37.1% in the top third, 32.3% in the mid third, and 10.8% in the low third (Table 3).

**Table 2.** The mean and median number of students and mean and median percentage of students’ grades on Standardized Letters of Evaluation (SLOE) for each program. (Median of 21 [interquartile range 15–30] SLOEs per program.)

	Mean number* (SD)	Median number* (IQR)	Mean percentage** (SD)	Median percentage** (IQR)
Honors/A	16.4 (22.7)	9.0 (2.0–20.9)	26.9% (0.2%)	23.0% (8%–40%)
High pass/B	18.5 (23.0)	12.1 (1.9–24.8)	30.7% (0.2%)	33.0% (8%–40%)
Pass/C	24.2 (32.8)	12.0 (3.3–33.3)	41.7% (0.4%)	35.0% (10%–70%)
Low pass/D	0.1 (1.4)	0.0 (0–0)	0.2% (0.0%)	0% (0%–0%)
Fail/F	0.2 (0.7)	0.0 (0–0)	0.3% (0.0%)	0% (0%–0%)

* The total number of students receiving a given grade by each program.

** The percentage of students receiving a given grade by each program.

*IQR,* interquartile range.

**Table 3.** The mean and median number of students and mean and median percentage of students’ rank-list positions on Standardized Letters of Evaluation (SLOE) for each program. (Median of 21 [IQR 15–30] SLOEs per program.)

	Mean number* (SD)	Median number* (IQR)	Mean percentage^**^ (SD)	Median percentage^**^ (IQR)
Top 10%	4.1 (2.9)	3.0 (2–5)	19.8% (0.1%)	16.4% (10.7%–25.2%)
Top third	8.6 (5.6)	7.0 (5–12)	37.1% (0.1%)	36.1% (27.8%–45.1%)
Mid third	8.0 (6.7)	7.0 (4–11)	32.3% (0.2%)	32.1% (25%–41.2%)
Low third	2.8 (3.3)	2.0 (0–4)	10.8% (0.1%)	8.8% (0%–17.3%)

* The total number of students receiving a given grade by each program.

** The percentage of students receiving a given grade by each program.

*IQR,* interquartile range.

## DISCUSSION

This study provides an updated representation of national trends in EM SLOE grade and rank distribution. Historically, significant emphasis has been placed on the SLOE grade and rank list. Current issues with the SLOE writing system include concerns ranging from inexperienced authors and non-standardized grading schemes to systematic grade inflation.

With the continued use of non-standardized grading schemes, it may appear this element of the SLOE provides little value. Our results show that while a majority of programs used the H/HP/P/F scheme, nearly 30% of programs favored other formats. Of those SLOEs using the P/F system, of which there were nearly 2,000 graded students, only one recorded a failing grade. With such an overwhelming predominance of passing grades, there may be more significance for a failing grade than a passing grade. Results from non-P/F programs show a nearly equal distribution of grades between Honors/A, High Pass/B, and Pass/C (31.6%, 35.6%, and 32.3%, respectively) with less than 1% receiving a grade of low pass or fail.

While SLOE 2.0 no longer contains a global assessment, the rank list remains a valued tool for differentiating applicants. Previous studies have demonstrated an improved spread of distribution over the past decade^2^ ^,^ ^4^; however, that trend may have become stagnant. Our data shows a nearly identical rank-list distribution to that of the 2016–2017 SLOE dataset, in which the top 10% contained 18%, the top third contained 37%, the mid third contained 35%, and the low third contained 10%, respectively (with our results showing 17.6%, 36.5%, 54.1%, and 11.8%, respectively).^4^ Whether this is a coincidence or evidence that the distribution of the rank list has truly stagnated remains unclear. Evaluators continue to rank very few applicants in the low third, representing an overly favorable evaluation of their rotating students.

## LIMITATIONS

There are several limitations that warrant consideration. First, this study was limited to a single year; future work should evaluate differences in grading trends over time. Additionally, the data was limited to self-report, and it is possible that some programs may not have accurately reported their grade and rank distribution for the prior year. While the majority of programs used the H/HP/P/F scale, some programs used alternate scales, which may not fully map to the more common H/HP/P/F scale. Moreover, we were limited to applicants who applied only to our two programs. Despite this limitation, we had a very high response rate and only missed 10 programs nationally. Finally, the rank-list reports perceived rank-list position but may not have reflected students’ actual position on the rank list.

## CONCLUSION

We determined the grading formats and grade, and rank-list distributions for EM programs during the 2022–2023 academic year. Most programs used the Honors/High Pass/Pass/Fail grading scheme, with the majority of students receiving Honors and High Pass, while 0.3% failed their rotation. Both grades and rank list demonstrated evidence of skewed distribution toward higher grades and rank-list position.

## Supplementary Information




