# Supplementary material for: Emergency Medicine Clerkship Grading Scheme, Grade, and Rank-List Distribution as Reported on Standardized Letters of Evaluation
Source: West J Emerg Med. 2024 Oct 29;26(1):66–9. doi: 10.5811/westjem.18687 (PMC11908520; doi:10.5811/westjem.18687)
Supplement: Supplementary file 1 [file wjem-26-66-s001.docx]

Appendix 1: “Other” grading schemes as reported by 20 programs.

| Grading Scheme | Number of Programs using grinding scheme  (% of total programs) |
| --- | --- |
| Honors, Pass, Fail | 13 (4.9%) |
| High Pass, Pass, Fail | 1 (0.4%) |
| High Honors, Honors, Pass, Fail (1/264), | 1 (0.4%) |
| Honors, High Satisfactory, Satisfactory, Low Satisfactory, Fail | 2 (0.8%) |
| Honors, A, A-, B+, B (1/264), | 1 (0.4%) |
| Honors, A,B,C, F (1/264), | 1 (0.4%) |
| High Distinction, Honors,Pass, Unsatisfactory (1/264). | 1 (0.4%) |
